# Neurological Phenotype and MRI Severity as Predictors of Duloxetine Response in Lumbar Spinal Stenosis: A Retrospective Cohort Study

**DOI:** 10.3390/jcm15103708

**Published:** 2026-05-12

**Authors:** Kazuya Honjoh, Arisa Kubota, Shuji Watanabe, Mikiko Kamitani, Yumiko Watanabe, Hideaki Nakajima

**Affiliations:** Department of Orthopaedics and Rehabilitation Medicine, University of Fukui Faculty of Medical Sciences, 23-3 Matsuoka Shimoaizuki, Eiheiji-cho, Yoshida-gun, Fukui 910-1193, Japan; kazuya@u-fukui.ac.jp (K.H.); akubota@u-fukui.ac.jp (A.K.); shujiw@u-fukui.ac.jp (S.W.); mikiko@u-fukui.ac.jp (M.K.); watayu@u-fukui.ac.jp (Y.W.)

**Keywords:** lumbar spinal stenosis, duloxetine, neuropathic pain, symptom-based pharmacotherapy

## Abstract

**Background/Objectives:** Lumbar spinal canal stenosis (LSS) is a major cause of neurological disability and is frequently accompanied by neuropathic pain (NeP). Duloxetine is widely used for NeP, but its clinical role in LSS and the determinants of treatment response remain unclear. This study aimed to identify predictors of duloxetine efficacy in LSS. **Methods:** We retrospectively analyzed 145 patients with LSS who received duloxetine for ≥3 months (median dose 40 mg) with at least 1-year follow-up. Patients were classified into those with radicular pain or cauda equina syndrome. Treatment response was assessed at 3 months. Stenosis severity was evaluated using the Schizas classification. Multivariable logistic regression analysis was performed to identify independent predictors of duloxetine response in patients with cauda equina syndrome. **Results:** Duloxetine was effective in 29.4% vs. 52.3% in radicular pain and cauda equina syndrome, respectively. Among patients with cauda equina syndrome, Schizas Grade D was more frequent in responders than non-responders and independently predicted duloxetine response. Nearly 90% of responders had been unresponsive to other NeP medications. A subset of patients with severe stenosis avoided surgery following duloxetine treatment. **Conclusions:** Duloxetine showed greater efficacy in cauda equina-dominant LSS compared with radicular pain. Paradoxically, more severe spinal canal stenosis tended to be associated with a higher likelihood of response. These findings suggest that neurological phenotype and radiological severity may influence duloxetine responsiveness and support a symptom- and imaging-guided pharmacological management for LSS, although these findings should be interpreted with caution and considered hypothesis-generating given the retrospective study design.

## 1. Introduction

Lumbar spinal stenosis (LSS) is a prevalent degenerative spinal disorder in aging societies and a major cause of activity-limiting neurological symptoms. In Japan, population-based epidemiological studies have found a prevalence of radiographically defined LSS of 9% to 13% in the general population and >20% in individuals aged ≥70 years [1]. Neuropathic pain (NeP) is common in spinal disorders [2] and more than half of patients with spinal diseases exhibit NeP components, with LSS accounting for about 56% of cases with NeP [3]. These findings indicate that LSS is a significant clinical and socioeconomic burden in super-aging societies. Persistent neuropathic symptoms are associated with reduced mobility, frailty progression, and increased healthcare utilization, emphasizing the importance of effective long-term pharmacological management.

NeP associated with spinal disorders is characterized by heterogeneous clinical manifestations, including radicular pain, neurogenic intermittent claudication, and cauda equina-related symptoms. Current international and Japanese clinical practice guidelines recommend gabapentinoids (pregabalin and mirogabalin) and serotonin–noradrenaline reuptake inhibitors (SNRIs), such as duloxetine, as first-line pharmacological treatments for NeP [4,5]. However, these recommendations are largely derived from evidence across diverse etiologies of NeP, including diabetic peripheral neuropathy and postherpetic neuralgia [6,7,8], and do not sufficiently account for disease- or symptom-specific differences in spinal disorders. Recent comparative evidence has highlighted the limited disease-specific basis for selecting among these agents. A 2024 systematic review and meta-analysis of direct head-to-head randomized controlled trials reported no consistent superiority between SNRIs, including duloxetine, and gabapentinoids across NeP, suggesting that treatment response may depend more on patient phenotype than on drug class alone [9]. In the current situation, selection of NeP medications in patients with LSS is often left to the discretion of physicians, leading to variability in treatment strategies and outcomes. In addition, interindividual variability in duloxetine response may partly reflect pharmacogenetic differences, particularly in CYP2D6- and CYP1A2-mediated metabolism, which influence duloxetine exposure and therapeutic response [10,11]. Although such pharmacogenetic findings have mainly been reported outside spinal disorders, they further support the need for individualized treatment strategies.

The gap between guidelines and real-world clinical practice suggests a need for a more individualized, symptom-based approach to pharmacotherapy in LSS. A patient-based retrospective study of NeP related to spinal disorders showed that treatment responses differed significantly among neurological symptoms [3]. Notably, duloxetine had a relatively favorable effect in patients with cauda equina syndrome, suggesting that the neurological phenotype may modify the treatment response. However, the clinical and radiological profiles most likely to benefit from duloxetine remain unclear. Therefore, identifying subgroups of patients with LSS who are more likely to respond to duloxetine is of clinical importance.

The aims of the present study are to examine the role of duloxetine in LSS and investigate differences in treatment efficacy based on clinical symptoms and imaging findings, with a focus on neurological symptom patterns and radiological characteristics. Identifying the clinical and radiological characteristics associated with a favorable response should allow definition of the position of duloxetine in pharmacological management of LSS.

## 2. Materials and Methods

### 2.1. Study Population

**Figure 1:**
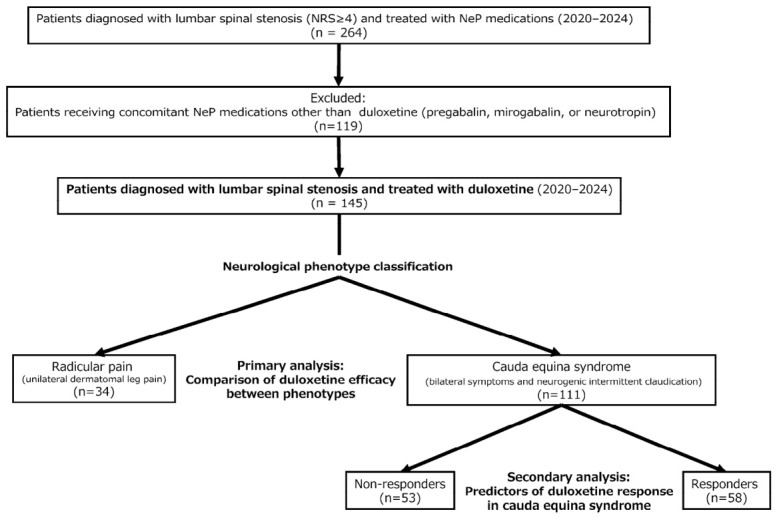
Flowchart of the study population. NRS: numerical rating scale; NeP: neuropathic pain.

Figure 1 shows a flowchart of the study population. Between 2020 and 2024, 264 patients were diagnosed with LSS who received a fixed dose of duloxetine for ≥3 months at our hospital. Duloxetine was prescribed for patients with persistent neuropathic leg symptoms despite at least 4 weeks of conservative management, including non-steroidal anti-inflammatory drugs and/or prior NeP medications, particularly in those with moderate to severe symptoms (numerical rating scale (NRS) ≥4) and clinically meaningful functional impairments, such as neurogenic claudication limiting walking capacity. In this study, patients receiving concomitant NeP medications other than duloxetine (pregabalin, mirogabalin, or neurotropin) were excluded to avoid confounding effects of multiple NeP agents. LSS was diagnosed based on a combination of characteristic clinical presentation and lumbar magnetic resonance imaging (MRI). Patients with prior spinal surgery, lumbar trauma, rheumatoid arthritis, destructive spondyloarthritis, neoplasm, infection, and lumbar disc herniation were also excluded from the study. Finally, 145 patients (92 males, 53 females; average age 73.5 years) were included in this study, and had a minimum follow-up period of one year. Patients were classified into those with radicular pain and those with cauda equina syndrome based on neurological symptom patterns at the time of initiation of duloxetine. Radicular pain was defined as unilateral dermatomal leg pain consistent with nerve root involvement. Cauda equina syndrome was defined by bilateral symptoms and neurogenic intermittent claudication accompanied by sensory disturbances. In cases presenting with mixed clinical features, classification was primarily determined by the presence of neurogenic intermittent claudication. Patients with intermittent claudication were categorized as having cauda equina syndrome, even when radicular pain was also present, because intermittent claudication is generally considered a hallmark symptom of cauda equina involvement in LSS. The study protocol was approved by the Human Ethics Review Committee of our University Medical Faculty and strictly followed the Clinical Research Guidelines of the Ministry of Health, Labor, and Welfare of the Japanese Government, in accordance with the Declaration of Helsinki. The patients were informed that data from the study would be submitted for publication, and gave their consent.

### 2.2. Outcome Assessment and Definition of Treatment Response

Treatment response was assessed three months after initiation of duloxetine at a stable dose. Duloxetine was initiated at 20 mg/day and titrated based on clinical response and tolerability. The dose was gradually increased to 40 or 60 mg/day, typically after an initial assessment period of 1–2 weeks, when symptom control was insufficient. In principle, dose escalation to at least 40 mg/day was performed prior to efficacy assessment in cases of insufficient response. However, in patients who demonstrated a clinically meaningful response at 20 mg/day, the same dose was maintained without escalation. Similarly, in patients who experienced adverse effects such as somnolence or loss of appetite but were able to continue treatment, dose escalation was not pursued if they preferred not to increase the dose, and treatment was continued at 20 mg/day. All patients were followed for a minimum of one year after treatment initiation to evaluate longitudinal clinical outcomes and subsequent treatment decisions. A NRS was used to evaluate leg pain intensity, with 0 representing no pain and 10 indicating the most severe pain ever experienced. Given the distinct pathophysiological mechanisms underlying radicular pain and cauda equina syndrome, response criteria were defined according to neurological phenotype: for radicular pain, responders were defined as those achieving a ≥50% reduction in NRS score from baseline; whereas for cauda equina syndrome, responders were those achieving a ≥50% reduction in NRS score and a ≥50% improvement in intermittent claudication [12,13]. All other patients were classified as non-responders.

### 2.3. Clinical and Radiological Variables

**Figure 2:**
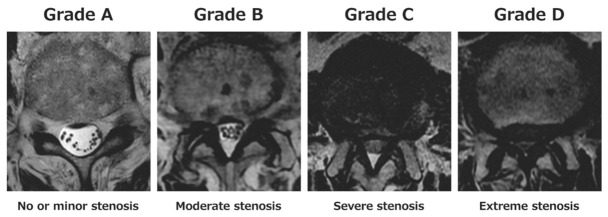
Schizas classification of lumbar spinal canal stenosis. Representative axial T2-weighted MRI showing Schizas Grades A–D. Grade A: clearly visible cerebrospinal fluid with dorsally arranged nerve rootlets; Grade B: rootlets occupying the dural sac, but individually identifiable; Grade C: a homogeneous gray signal without recognizable rootlets, but with preserved posterior epidural fat; and Grade D: complete obliteration of both rootlets and posterior epidural fat.

Clinical variables, including age, sex, body mass index (BMI), baseline NRS, diabetes mellitus, low back pain, and surgical treatment during follow-up, were obtained from medical records. Treatment-related variables included the final dose of duloxetine (20, 40 or 60 mg/day) and use of other NeP medications before duloxetine initiation. Lumbar MRI was performed in all patients, and spinal canal stenosis was evaluated on axial T2-weighted images using the Schizas classification (Figure 2) [14]. In this system, Grade A is defined as no or minor stenosis with clearly visible cerebrospinal fluid (CSF) in the dural sac; Grade B is moderate stenosis with rootlets occupying the dural sac, but still individually identifiable; Grade C is severe stenosis with no recognizable rootlets and a homogeneous gray signal in the dural sac; and Grade D is extreme stenosis with obliteration of both rootlets and posterior epidural fat. Spinal segments graded as Schizas Grade C or D were defined as stenotic segments. In patients with multilevel stenosis, the segment with the most severe stenosis was used as the index level for analysis. Treatment response was analyzed in relation to the Schizas grade at the index level.

### 2.4. Statistical Analysis

Continuous variables are presented as medians with interquartile ranges (IQRs) and compared by Mann–Whitney U test. Categorical variables are presented as numbers and percentages and compared by chi-square test. A two-sided *p*-value < 0.05 was considered statistically significant. Variables with significance in univariate analysis and some patient backgrounds were subsequently included in a multivariate logistic regression model. To identify independent predictors of duloxetine response in patients with cauda equina syndrome, odds ratios (ORs) with 95% confidence intervals (CIs) were calculated. All statistical analyses were performed using EZR (Saitama Medical Center, Jichi Medical University, Saitama, Japan) and GUI for R (Version 1.61, The R Foundation for Statistical Computing, Vienna, Austria) [15].

### 2.5. Generative AI Statement

The authors declare that Gen AI was used in creation of this manuscript. OpenAI’s ChatGPT (GPT-5.2) was used only for improving grammar, readability, and language of the Abstract, Introduction, and Discussion sections. After using this tool, the corresponding author (H.N.) and first author (K.H.) reviewed and edited the content as needed and take full responsibility for the content of the publication. No data analysis, interpretation, or scientific conclusions were generated by AI tools.

## 3. Results

### 3.1. Demographics and Comparison of Duloxetine Efficacy for Neurological Phenotypes

A total of 145 patients were included in the analysis, including 34 with radicular pain and 111 with cauda equina syndrome. The final duloxetine dose was 20 mg/day in 67 patients, 40 mg/day in 59 patients, and 60 mg/day in 19 patients (median dose 40 mg/day). Duloxetine was effective in 29.4% of patients with radicular pain and 52.3% of patients with cauda equina syndrome (*p* = 0.032). Age, sex, BMI, baseline NRS, prevalence of diabetes mellitus and dose of duloxetine did not differ significantly between the two groups (Table 1). Among patients who continued treatment but remained on 20 mg/day because of adverse effects limiting dose escalation, 7 patients had radicular pain and 18 had cauda equina syndrome. The most common adverse effects were somnolence (n = 14), nausea and/or appetite loss (n = 8), and dry mouth (n = 3).

### 3.2. Predictors of Duloxetine Response in Patients with Cauda Equina Syndrome

The clinical and radiological factors associated with duloxetine response in patients with cauda equina syndrome are summarized in Table 2. Demographic variables and clinical features did not differ significantly between responders (52.3%) and non-responders. Of note, severe spinal canal stenosis (Schizas Grade D) was significantly more frequent in responders (62.1% vs. 41.5%, *p* = 0.048), whereas surgical intervention was significantly more common in non-responders (45.3% vs. 0%, *p* < 0.001). During follow-up, 24 patients (21.6%) required surgical decompression. Among non-responders, the proportion of cases undergoing surgery differed according to MRI severity. More patients with Schizas Grade D stenosis required surgery compared to those with Schizas Grade C stenosis (13/22 (59.1%) vs. 11/31 (35.5%)).

### 3.3. Prior Use of Neuropathic Pain Medication

Among duloxetine responders, 52 patients (89.7%) had been treated with other Nep medications prior to duloxetine initiation (Table 3). The common prior medications were pregabalin (51.7%), neurotropin (36.2%), and mirogabalin (19.0%). Typical dose ranges before switching to duloxetine were 150–300 mg/day for pregabalin, 20–30 mg/day for mirogabalin, and standard approved dosing for neurotropin (16 units). These agents had failed to provide clinically meaningful symptom relief, prompting a switch to duloxetine.

### 3.4. Multivariable Logistic Regression Analysis of Predictors of Treatment Efficacy

Multivariable logistic regression analysis was performed to identify independent predictors of duloxetine efficacy in patients with cauda equina syndrome (Table 4). Severe spinal canal stenosis, defined as Schizas Grade D, was independently associated with a higher likelihood of response to duloxetine (odds ratio [OR], 2.75; 95% confidence interval [CI], 1.04–7.28; *p* = 0.042). In contrast, age (OR, 0.96 per 1-year increase; 95% CI, 0.91–1.01; *p* = 0.12), male sex (OR, 0.98; 95% CI, 0.40–2.37; *p* = 0.96), number of stenotic segments (OR, 1.06 per segment; 95% CI, 0.70–1.60; *p* = 0.80), and duloxetine dose (40 mg or 60 mg vs. 20 mg) were not significantly associated with treatment response.

## 4. Discussion

This study investigated the clinical and radiological characteristics associated with the therapeutic efficacy of duloxetine in patients with LSS, with a focus on neurological symptom patterns. The principal findings are as follows: (1) duloxetine was significantly more effective in patients with cauda equina syndrome than in those with radicular pain; (2) in cauda equina syndrome cases, those with more severe spinal canal stenosis paradoxically had a higher likelihood of responding to duloxetine. These findings provide novel insights into the symptom- and pathology-specific efficacy of duloxetine in LSS and have important implications for individualized pharmacological management of NeP associated with degenerative spinal disorders.

Previous clinical investigations have shown that the therapeutic response to NeP medications differs according to pain phenotypes and neurological symptom patterns. In a patient-based study of NeP related to spinal disorders, duloxetine showed a relatively favorable effect for cauda equina syndrome, whereas its efficacy was limited for spinal cord-related pain [3]. The present study extends these findings by focusing on LSS and showing that duloxetine responsiveness is strongly associated with cauda equina-dominant symptomatology, rather than radicular pain. The difference in duloxetine efficacy between these conditions may be explained by distinct pathophysiological mechanisms. Radicular pain is primarily caused by focal nerve root compression and inflammation, whereas cauda equina syndrome is characterized by multilevel compression and ischemia of the cauda equina, often accompanied by bilateral symptoms, sensory disturbances, and neurogenic intermittent claudication [16,17]. Duloxetine is a SNRI that enhances descending inhibitory pathways in the central nervous system and modulates pain processing at the spinal and supraspinal levels [18]. Therefore, duloxetine may be particularly effective for conditions in which central sensitization and diffuse neural dysfunction contribute to symptom generation, as observed in cauda equina-dominant LSS.

One intriguing finding of the study is that patients with more severe spinal canal stenosis, as assessed by the Schizas classification, were more likely to respond to duloxetine. This paradoxical association contrasts with the conventional assumption that severe mechanical compression is less responsive to pharmacological therapy. Several potential mechanisms may explain this phenomenon. Severe cauda equina compression may induce more pronounced neuropathic mechanisms, including central sensitization and alterations in monoaminergic pathways. Such central sensitization and plastic changes in descending inhibitory systems have been recognized as key contributors to chronic NeP [6,18]. Central sensitization is defined as an increased responsiveness of nociceptive neurons within the central nervous system and is increasingly recognized as a key mechanism underlying chronic NeP and the dissociation between structural pathology and symptom severity [19]. In conditions involving diffuse and sustained afferent input, such as multilevel cauda equina compression, persistent nociceptive signaling may facilitate maladaptive spinal and supraspinal processing, leading to amplification of pain perception independent of the degree of structural compression. This framework may help explain why patients with more severe stenosis do not necessarily experience poorer pharmacological response and, paradoxically, may demonstrate greater responsiveness to centrally acting agents. Descending monoaminergic pathways, particularly noradrenergic and serotonergic systems, play a crucial role in modulating spinal nociceptive transmission. Duloxetine enhances these descending inhibitory pathways and modulates pain processing at the spinal and supraspinal levels [20]. Serotonin–noradrenaline reuptake inhibitors increase synaptic availability of these neurotransmitters and strengthen descending inhibitory control over nociceptive transmission [19], thereby counteracting central sensitization-related pain mechanisms. Emerging evidence also suggests that duloxetine may attenuate pain associated with central sensitization, further supporting its potential efficacy in conditions characterized by diffuse neural dysfunction rather than focal nerve compression. Therefore, duloxetine may preferentially target centrally mediated neuropathic mechanisms that are more prominent in cauda equina-dominant pathology than in focal radicular pain. Experimental studies have suggested that duloxetine exerts analgesic effects in models of cauda equina compression by modulating spinal monoaminergic systems, supporting the biological plausibility of our findings [21]. In these models, gabapentinoid-related molecular targets are less prominently upregulated, whereas descending inhibitory mechanisms play a more critical role in pain modulation. Taken together, these findings suggest that the paradoxically better response to duloxetine observed in patients with severe stenosis may reflect a greater contribution of centrally mediated pain mechanisms and impaired descending inhibitory control, rather than a direct beneficial effect of anatomical severity itself. These findings imply that NeP associated with cauda equina dysfunction may be more responsive to centrally acting agents such as duloxetine than to peripheral nerve-targeted therapies.

Another clinically relevant observation is that nearly 90% of duloxetine responders had previously received other NeP medications, including gabapentinoids and neurotropin, without sufficient analgesic effects. Current clinical guidelines recommend gabapentinoids and SNRIs as first-line pharmacological treatments for NeP; however, they do not provide specific recommendations for LSS based on neurological symptom patterns or radiological severity. Gabapentinoids act primarily by modulating voltage-gated calcium channels at the presynaptic level [22], whereas duloxetine targets descending inhibitory pathways through serotonin and noradrenaline reuptake inhibition. These distinct mechanisms of action suggest that duloxetine may provide additive or complementary analgesic effects in patients who are refractory to gabapentinoids. Our findings suggest that duloxetine may play a central role in the pharmacological management of cauda equina-dominant LSS. Given its favorable efficacy in this phenotype, duloxetine could be considered as an early-line therapeutic option, rather than merely a second-line agent. Neurotropin, which also acts on descending inhibitory pathways and has a favorable safety profile, may reasonably be used as initial treatment [23,24]; however, our results indicate that prompt switching to duloxetine when symptom control is insufficient may be a more efficient strategy. Importantly, many patients with severe stenosis (Schizas Grade D) did not respond to duloxetine and ultimately required surgery, but a subset of patients with similarly severe stenosis responded favorably and avoided surgical intervention. These observations suggest that imaging severity alone does not determine pharmacological responsiveness and that consideration of duloxetine therapy before definitive surgical decision-making may be justified, even in anatomically severe cases. This symptom-based approach to pharmacotherapy may contribute to more rational and individualized treatment strategies for LSS.

Several limitations of the study should be acknowledged. First, the retrospective design may have introduced selection bias and unmeasured confounding factors. Second, the definition of treatment effectiveness was based on clinical assessment, including the NRS, rather than standardized outcome measures in all cases. The NRS is inherently subjective, which could introduce measurement bias, and objective functional measures were not systematically assessed. In addition, electrophysiological and laboratory evaluations were not systematically performed, and psychiatric comorbidities were not formally assessed, which may have allowed unrecognized peripheral neuropathy, including diabetic polyneuropathy, vascular disease, or psychological factors to influence the results. Third, the sample size was relatively small, particularly in subgroup analyses, which may limit the generalizability of the findings. Fourth, the study was conducted at a single center, and external validation in multicenter cohorts is warranted. Fifth, causal relationships between radiological severity and duloxetine responsiveness cannot be definitively established based on the observational design. Finally, duloxetine is not formally approved for the treatment of LSS or NeP as a specific indication. The approved indications include major depressive disorder, diabetic neuropathic pain, fibromyalgia, chronic low back pain, and pain associated with osteoarthritis. Consequently, the use of duloxetine for LSS in this study constitutes off-label treatment, and this regulatory context should be taken into account when interpreting the results. Despite these limitations, the study provides important evidence supporting a symptom- and pathology-oriented approach to pharmacological management of LSS. Prospective studies with standardized outcome measures and larger sample sizes are needed to confirm the predictive value of neurological symptom patterns and radiological severity for duloxetine responsiveness. Mechanistic studies of the interaction between cauda equina pathology and monoaminergic pain modulation may further establish the biological basis of the observed therapeutic effects.

## 5. Conclusions

Duloxetine showed significantly greater efficacy in patients with cauda equina-dominant LSS than in those with radicular pain. Paradoxically, patients with more severe spinal canal stenosis had a higher likelihood of responding to duloxetine, and many responders had experienced insufficient analgesia with other NeP medications. These findings may help inform clinical decision-making and support the development of symptom-based pharmacological strategies for NeP. In patient with cauda equina-dominant LSS, a trial of duloxetine may be considered before proceeding to surgery. The present findings should be interpreted as hypothesis-generating, and further prospective, adequately powered studies are required to confirm these findings and clarify their clinical implications.

## Figures and Tables

**Table 1:** Comparison of patients with radicular pain and cauda equina syndrome.

Item	Radicular Pain	Cauda Equina Syndrome	*p*-Value
Number of patients, n (%)	34	111	
Background			
Age (years), median [IQR]	73.0 [69.0, 76.8]	76.0 [69.0, 80.0]	0.13
Sex (male/female), n (%))	19 (55.9%)/15 (44.1%)	73 (65.8%)/38 (34.2%)	0.40
BMI (kg/m^2^), median [IQR]	24.7 [22.9, 26.8]	24.4 [22.3, 26.7]	0.78
Baseline NRS	8.0 [6.0, 9.0]	7.0 [6.0, 8.0]	0.49
Diabetes, n (%)	11 (32.4%)	29 (27.9%)	0.78
Dose of duloxetine			
20 mg	14 (41.2%)	53 (47.7%)	0.40
40 mg	17 (50.0%)	42 (37.8%)	
60 mg	3 (8.8%)	16 (14.4%)	
Effectiveness of duloxetine	10 (29.4%)	58 (52.3%)	0.032 *

IQR: interquartile range; BMI: body mass index; NRS: numerical rating scale, * *p* < 0.05.

**Table 2:** Associations of clinical and radiological factors with duloxetine response in patients with cauda equina syndrome.

Item	Non-Responders	Responders	*p*-Value
Number of patients, n (%)	53 (47.7%)	58 (52.3%)	
Background			
Age (years), median [IQR]	77.0 [70.5, 79.5]	74.0 [68.3, 79.8]	0.42
Sex (male/female), n (%))	36 (67.9%)/17 (32.1%)	37 (63.8%)/21 (36.2%)	0.80
BMI (kg/m^2^), median [IQR]	24.6 [23.2, 26.8]	23.8 [20.9, 25.6]	0.12
Baseline NRS	7.0 [6.0, 8.0]	7.0 [5.3, 8.0]	0.89
Diabetes, n (%)	15 (30.6%)	14 (25.5%)	0.71
Low back pain, n (%)	25 (47.2%)	26 (44.8%)	0.96
Imaging findings			
Listhesis, n (%)	15 (28.3%)	15 (25.9%)	0.94
Stenosis segments, median [IQR]	2.0 [1.0, 2.0]	2.0 [1.0, 3.0]	0.27
Schizas classification			
Grade C, n (%)	31 (58.5%)	22 (37.9%)	0.048 *
Grade D, n (%)	22 (41.5%)	36 (62.1%)	
Dose of duloxetine			
20 mg, n (%)	25 (47.2%)	28 (48.3%)	0.16
40 mg, n (%)	17 (32.1%)	25 (43.1%)	
60 mg, n (%)	11 (20.8%)	5 (8.6%)	
Surgery, n (%)	24 (45.3%)	0 (0%)	<0.001 *

IQR: interquartile range; BMI: body mass index, * *p* < 0.05.

**Table 3:** Use of prior NeP medications that were ineffective in patients with cauda equina syndrome who were responsive to duloxetine.

Other NeP Medications Used Before Duloxetine (Ineffective for Cauda Equina Syndrome)			
Number of patients, n (%)	52 (89.7%)		
	Neurotropin	Pregabalin	Mirogabalin
Number of patients per drug, n (%)	21 (36.2%)	30 (51.7%)	11 (19.0%)

**Table 4:** Multivariable logistic regression analysis of potential predictors of duloxetine efficacy in patients with cauda equina syndrome.

Variable	OR	95% CI	*p* Value
Age (per 1-year increase)	0.96	0.91–1.01	0.12
Male sex	0.98	0.40–2.37	0.96
Number of stenotic segments (per segment)	1.06	0.70–1.60	0.80
Severe stenosis (Schizas Grade D)	2.75	1.04–7.28	0.042 *
Duloxetine 40 mg (vs. 20 mg)	1.21	0.52–2.82	0.66
Duloxetine 60 mg (vs. 20 mg)	0.45	0.13–1.56	0.21

OR, odds ratio; 95% CI, 95% confidence interval; * *p* < 0.05.

## Data Availability

Data generated and analyzed during this study are included in this published article. Data and materials are available from the corresponding author subject to reasonable request and subject to the ethical approvals in place and materials transfer agreements.

## Abbreviations

LSS	Lumbar spinal stenosis
NeP	Neuropathic pain
SNRI	Serotonin–noradrenaline reuptake inhibitors
NRS	Numerical rating scale
MRI	Magnetic resonance imaging
BMI	Body mass index
CSF	Cerebrospinal fluid
IQRs	Interquartile ranges
ORs	Odds ratios
CIs	Confidence intervals
